# Healthcare-facility-based SARS-CoV-2 genomic surveillance in Brazil: experience from the global action in healthcare network

**DOI:** 10.1128/spectrum.02390-25

**Published:** 2026-07-02

**Authors:** Flávia Roberta Brust, Mateus Swarovsky Helfer, Beatriz Arns, Eunice Beatriz Martin Chaves, Fabio Fernandes Dantas Filho, Gabriela Luchiari Tumioto Giannini, Giovana Bristot, Karine Lorenzen Molina, Marcelo Bitelo da Silva, Tiago Finger Andreis, Vlademir Vicente Cantarelli, Alexandre Prehn Zavascki

**Affiliations:** 1 Health Security Partners 828060 , Washington, DC, USA; 2 Infectious Disease Service, Hospital de Clínicas de Porto Alegre 37895, Porto Alegre, Rio Grande do Sul, Brazil; 3 Infectious Disease and Infection Control Service, Hospital Moinhos de Vento 156417, Porto Alegre, Rio Grande do Sul, Brazil; 4 Infection Control Service, Hospital de Clínicas de Porto Alegre, Porto Alegre, Rio Grande do Sul, Brazil; 5 Occupational Medicine Service, Hospital de Clínicas de Porto Alegre 37895, Porto Alegre, Rio Grande do Sul, Brazil; 6 Postgraduate Program in Pneumological Sciences, Universidade Federal do Rio Grande do Sul 28124, Porto Alegre, Rio Grande do Sul, Brazil; 7 Universidade do Vale do Rio dos Sinos 37906, São Leopoldo, Rio Grande do Sul, Brazil; 8 Laboratório de Genética e Biologia Molecular, Hospital Moinhos de Vento 156417, Porto Alegre, Rio Grande do Sul, Brazil; 9 Internal Bed Regulation Committee, Hospital de Clínicas de Porto Alegre 37895, Porto Alegre, Rio Grande do Sul, Brazil; 10 Unimed Vale dos Sinos, Novo Hamburgo, Rio Grande do Sul, Brazil; 11 Universidade Federal de Ciências da Saúde de Porto Alegre 117303, Porto Alegre, Rio Grande do Sul, Brazil; 12 Department of Internal Medicine, Universidade Federal do Rio Grande do Sul 28124, Porto Alegre, Rio Grande do Sul, Brazil; Barnard College, Columbia University, New York, New York, USA

**Keywords:** SARS-CoV-2, healthcare workers, Omicron, Brazil, healthcare-facility-based genomic surveillance, health personnel, global heath

## Abstract

Genomic sequencing is essential to effectively monitor the SARS-CoV-2 evolution and spread of its lineages. Healthcare-facility-based SARS-CoV-2 genomic surveillance has been proposed as a valuable strategy, considering the characteristics of its target population. As part of the Centers for Disease Control and Prevention’s Global Action in Healthcare Network program, this study aimed to describe the distribution and frequency of SARS-CoV-2 lineages in two tertiary-care hospitals in Brazil, where the genomic sequencing capacity is limited. Whole-genome sequencing of SARS-CoV-2 samples obtained from 993 healthcare workers (75.4%) and inpatients (24.6%) was analyzed between February 2023 and August 2024. In total, 113 distinct lineages were identified. Notably, we observed a temporal replacement of predominant lineages corresponding to three distinct epidemic waves: the first wave dominated by XBB.1.5 and XBB.2.3 (February 2023 to June 2023), the second by GK.1.1 and JD.1.1 (September 2023 to December 2023), and the third by JN.1 and JN.1.9 (January 2024 to April 2024). JN.1.9 was the only lineage with a significantly higher prevalence among healthcare workers compared to inpatients. Additionally, we identified cases of co-infection with genetically distinct variants, underscoring the potential for healthcare-based monitoring to capture events relevant to viral evolution. Overall, our findings were consistent with those observed across Brazil, suggesting that this strategy may be valuable for SARS-CoV-2 genomic surveillance. They also indicate a clear temporal pattern of lineage replacement, reflecting successive waves driven by emerging variants and rapid global dissemination.

**IMPORTANCE:**

Genomic surveillance of SARS-CoV-2 remains essential for identifying emerging variants with increased transmissibility, immune escape, or pathogenicity. While most genomic surveillance efforts focus on community-based sampling, a healthcare-facility-based strategy may offer a complementary approach. In this study, we describe SARS-CoV-2 lineage dynamics over an 18-month period among healthcare workers and hospitalized patients in southern Brazil. Our findings align closely with regional and national trends, supporting the value of healthcare-facility-based SARS-CoV-2 genomic surveillance for documenting the local genomic landscape and demonstrating the feasibility and value of this approach in settings with limited genome sequencing capacity. Additionally, this approach may be applicable to other respiratory viruses in healthcare settings; however, further studies would be needed to confirm this.

## OBSERVATION

The course of the COVID-19 pandemic has been significantly shaped by the emergence of new SARS-CoV-2 variants with enhanced transmissibility due to genetic mutations (1). Early in the pandemic, each variant of concern (Alpha, Beta, Gamma, and Delta) was associated with a distinct, sequential wave of infections. During these periods, a single variant typically dominated, with limited co-circulation of other variants (1). Since the emergence of the Omicron variant (B.1.1.529) in November 2021 (2), however, the viral landscape has shifted. Numerous Omicron sublineages have emerged rapidly, leading to frequent but relatively short waves of infection, often characterized by increased transmissibility and immune escape (3).

To effectively track and respond to the evolution of SARS-CoV-2, robust genomic surveillance is critical. A key consideration is the strategic selection of target populations for sequencing (4). Healthcare workers (HCWs) and hospitalized patients have been identified as important sentinel groups for healthcare-facility-based SARS-CoV-2 genomic surveillance (5, 6). This approach offers multiple advantages: HCWs usually have access to testing facilitated and serve as a link between healthcare settings and the community (6, 7), and their typically high vaccination coverage enables the assessment of immune escape and breakthrough infections (8, 9). Inpatients, on the other hand, often represent a higher-risk population and may be more likely to represent infections by variants exhibiting increased pathogenicity (5, 7).

The Global Action in Healthcare Network (GAIHN), coordinated by the U.S. Centers for Disease Control and Prevention, is a multinational consortium focused on addressing emerging infectious disease threats in healthcare settings (10). In November 2022, GAIHN initiated SARS-CoV-2 genomic surveillance among HCWs and inpatients. The first results from Brazil, covering the period from November 2022 to January 2023, have been published (11). This report presents complementary data from Brazil, where surveillance continued through August 2024.

Genomic surveillance was conducted between February 2023 and August 2024 at two tertiary-care hospitals in the state of Rio Grande do Sul, Brazil. HCWs and inpatients aged 18 years or older who tested positive for SARS-CoV-2 by real-time reverse transcription PCR (RT-PCR) or rapid antigen test were eligible for inclusion. Respiratory samples were obtained using either oropharyngeal or nasopharyngeal swabs. For participants who initially tested positive by rapid antigen test, a follow-up swab was collected to confirm infection by RT-PCR (12). Only specimens with a cycle threshold (Ct) ≤30 for any gene target were submitted for whole-genome sequencing (see Supplemental material). Demographic data, vaccination history (number of doses), and symptom information were obtained by participant interview. Statistical analysis was conducted using the software R version 4.5.2. The study protocol was approved by the Institutional Review Boards of both hospitals (CAAE: 59038722.0.1001.5330), and informed consent was obtained from all participants.

Among 1,809 individuals who tested positive, 659 (36.4%) did not provide consent and were excluded. An additional 142 (7.8%) were excluded due to Ct value >30. Thirteen participants tested positive by rapid antigen test but negative by RT-PCR, and two specimens yielded low coverage, preventing lineage determination. In total, 993 specimens were successfully sequenced, including 749 (75.4%) from HCWs and 244 (24.6%) from inpatients (Fig. S1). Participant characteristics are presented in Table 1.

**TABLE 1:** Demographic and clinical characteristics of healthcare workers and inpatients included in SARS-CoV-2 genomic surveillance, February 2023–August 2024

Variables	Total *n* = 993 (%)	Inpatients *n* = 244 (%)	Healthcare workers *n* = 749 (%)
Female	722 (72.7)	124 (50.8)	598 (79.8)
Median age (IQR)		67.9 (56.3–77.4)	41.0 (32.4–48.7)
Previous infection	666 (67.1)	98 (40.2)	568 (75.8)
Symptoms description^*a*^			
Asymptomatic	17 (1.7)	17 (7.0)	0 (0)
Mild illness	895 (90.1)	146 (59.8)	749 (100.0)
Moderate illness	59 (5.9)	59 (24.2)	0 (0)
Severe illness	7 (0.7)	7 (2.9)	0 (0)
Critical illness	13 (1.3)	13 (5.3)	0 (0)
Unknown	2 (0.2)	2 (0.8)	0 (0)
Vaccinated	984 (99.1)	236 (96.7)	748 (99.9)
1 Dose	4 (0.4)	1 (0.4)	3 (0.4)
2 Doses	68 (6.8)	31 (12.7)	37 (4.9)
3 Doses	203 (20.4)	55 (22.5)	148 (19.8)
4 Doses	362 (36.5)	92 (37.7)	270 (36.0)
5 Doses	347 (34.9)	57 (23.4)	290 (38.7)
Not vaccinated	9 (0.9)	8 (3.3)	1 (0.1)
COVID-19 hospitalization	81 (8.2)	81 (33.2)	0

^*a*^ Classification according to clinical spectrum of SARS-CoV-2 infection—United States National Institute of Health (NIH).

**Fig 1:**
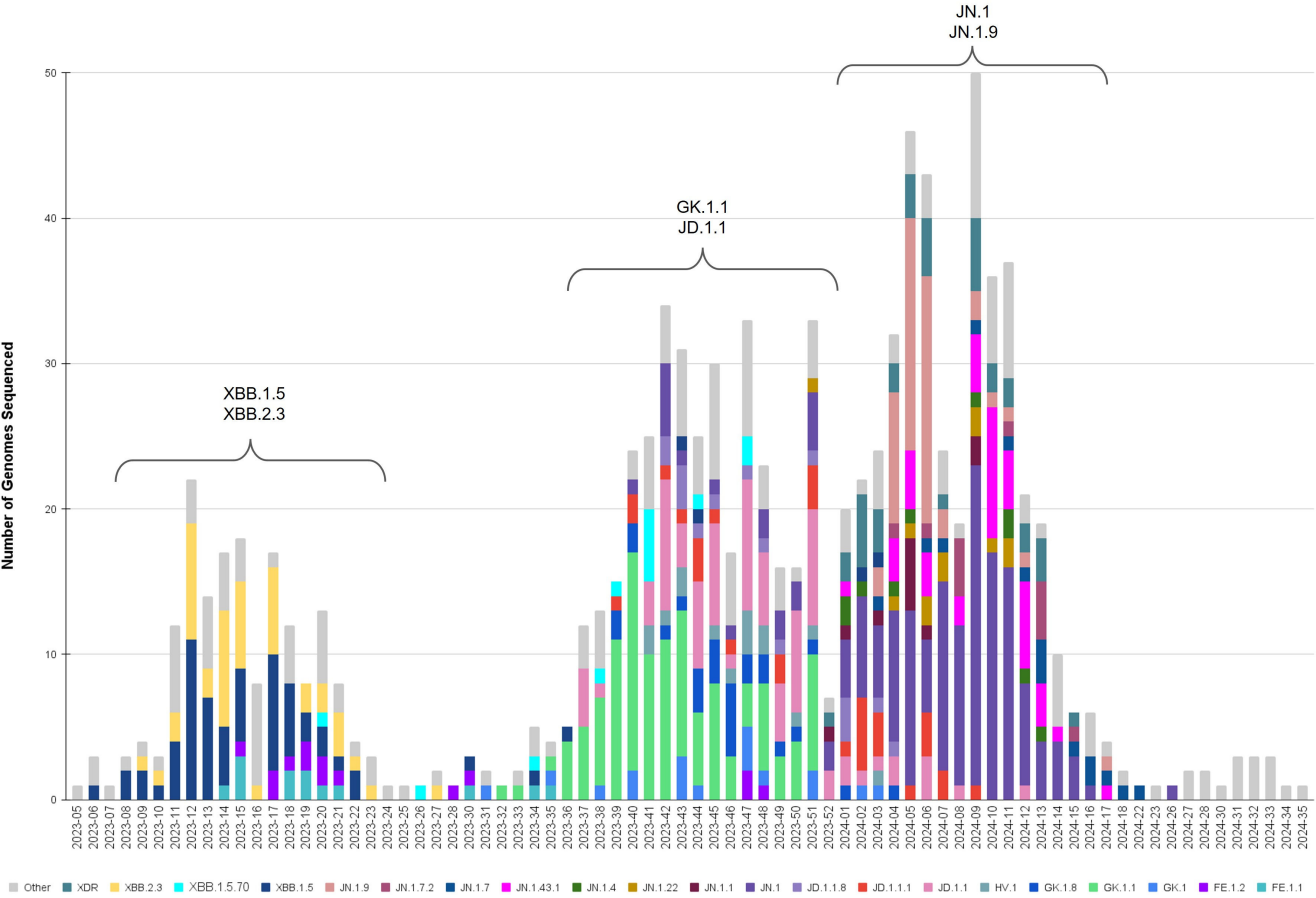
Distribution of SARS-CoV-2 lineages identified in healthcare workers and inpatients from two hospitals in Porto Alegre, Southern Brazil, February 2023 to August 2024 (*n* = 993 specimens). “Other” includes lineages with a prevalence of less than 1% over the study period.

A total of 113 distinct Omicron lineages were identified during the study period (Fig. 1; Table S1). The most frequently detected lineages were JN.1 (*n* = 162, 16.3%), GK.1.1 (*n* = 115, 11.6%), and JD.1.1 (*n* = 80, 8.1%).

Among the most frequent SARS-CoV-2 lineages, JN.1.9 was significantly more prevalent among HCWs than inpatients (6.4% vs 1.6%; adjusted *P* = 0.025). No other lineage showed statistically significant differences after correction for multiple comparisons (Fig. 1; Table S2).

Three distinct waves of infection were observed over the course of surveillance. The first wave (February–June 2023) was characterized by the predominance of XBB.1.5 and XBB.2.3. The second wave (September–December 2023) was dominated by GK.1.1 and JD.1.1. The third wave (January–April 2024) had JN.1 and JN.1.9 as the most common circulating lineages (Fig. 1).

During the first half of 2023, XBB lineages, specifically XBB.1.5 and XBB.2.3, replaced BQ.1 and BE.9, which had been the predominant lineage identified in our earlier study (11). According to the WHO classification, in March 2023, the Omicron subvariant XBB.1.5 was designated as a variant of interest (VOI), and in May 2023, XBB.2.3 was added to the list of variants under monitoring (13, 14). The “X-” lineages are recombinant lineages derived from previous variants of concern; in the case of XBB, recombination occurred between BA.2.10.1.1 and BA.2.75 Omicron sublineages. These recombinations enhanced immune escape capabilities and competitive fitness, allowing them to outcompete other circulating Omicron variants (15).

Data from the COVID-19 Fiocruz Genomic Surveillance Network (https://www.genomahcov.fiocruz.br/dashboard-en/) indicate that XBB.1.5* and FE.1* were the most frequently reported lineages in Brazil during the first 5 months of 2023 (Table S3). In our data, when FE.1.1 and FE.1.2 are grouped together, they rank among the three most prevalent lineages from April to August 2023. A similar distribution of circulating variants was reported in a separate study from Rio Grande do Sul (16). However, in contrast to our findings, XBB.2.3 appeared at a lower frequency in nationwide data based on a non-targeted population (17).

By August and September 2023, GK.1.1 (a descendant of lineage XBB.1.5.70) and JD.1.1 (a descendant of lineage XBB.1.5.102) began to emerge as the most common lineages in our population (18). These trends were also observed at the national level in Brazil (17). XBB-descended lineages remained dominant until October 2023 but were gradually displaced by descendants of BA.2.86, a VOI designated in November 2023 (19). These new lineages are phylogenetically distinct from earlier Omicron XBB variants (20).

In October 2023, we first detected the JN.1 lineage, which rose to dominance in January 2024, followed by JN.1.9 and JN.1.43, all descendants of BA.2.86. This pattern was consistent across all regions of Brazil (17, 21). These lineages remained dominant until April 2024, after which case numbers declined substantially through August.

Co-infection with two genetically distinct SARS-CoV-2 lineages was identified in two inpatients: one with BQ.1 + XBB.1 and another with BQ.1.1 + XBB.1.5. Such co-infections may contribute to viral recombination and the emergence of novel variants, highlighting the ongoing importance of genomic surveillance (22).

This study has several limitations. It is a descriptive study conducted in only two healthcare centers in a single city, potentially limiting the generalizability of the findings. Although healthcare-facility-based surveillance can be effective in detecting variants with immune escape or increased pathogenicity, it may be less sensitive to identifying lineages that are highly transmissible but cause milder illness or predominantly affect subgroups such as unvaccinated individuals. Furthermore, a considerable number of samples were excluded because participants did not consent or could not be contacted; however, we do not believe this substantially influenced the observed lineage frequency distribution, as there is no clear reason to expect that specific lineages would preferentially infect these individuals. In addition, 7.8% of the individuals who tested positive for SARS-CoV-2 had high Ct values (>30), which could represent lineages with different characteristics; this cut-off was selected to minimize failures related to low-quality sequences.

While most genomic surveillance efforts focus on community-based sampling, healthcare-facility-based strategies offer a complementary approach for monitoring SARS-CoV-2 circulation within hospital settings. Our findings demonstrate that the observed lineage distribution is comparable to reports from both the state of Rio Grande do Sul and national data, suggesting that this strategy can serve as a valuable complement to broader SARS-CoV-2 surveillance efforts. Additionally, healthcare-facility-based genomic surveillance might allow earlier detection of clinically significant variants; however, this possibility should be investigated in future studies using this surveillance strategy.

## Data Availability

All SARS-CoV-2 sequences were submitted to GISAID (23) for public access and lineage classification. All genome sequences and associated metadata generated and analyzed in this study are available through GISAID’s EpiCoV database and are listed in Table S3.

## SUPPLEMENTAL MATERIAL

Supplemental material spectrum.02390-25-s0001.docx Tables S1 to S3; Fig. S1.

ASM does not own the copyrights to Supplemental Material that may be linked to, or accessed through, an article. The authors have granted ASM a non-exclusive, world-wide license to publish the Supplemental Material files. Please contact the corresponding author directly for reuse.
