# Supplementary material for: Healthcare-facility-based SARS-CoV-2 genomic surveillance in Brazil: experience from the global action in healthcare network
Source: Microbiol Spectr. 2026 Jul 2;14(8):e02390-25. doi: 10.1128/spectrum.02390-25 (PMC13436415; doi:10.1128/spectrum.02390-25)
Supplement: Supplementary file 1 [file spectrum.02390-25-s0001.docx]

Healthcare-Facility-Based SARS-CoV-2 Genomic Surveillance in Brazil: Experience from the Global Action in Healthcare Network

Flávia Roberta Brust,^1*^ Mateus Swarovsky Helfer,^2^ Beatriz Arns,^2,10^ Eunice Beatriz Martin Chaves^3^, Fabio Fernandes Dantas Filho^3,4^, Gabriela Luchiari Tumioto Giannini^5^, Giovana Bristot^5^, Karine Lorenzen Molina^6^, Marcelo Bitelo da Silva,^7,8^ Tiago Finger Andreis,^5^ Vlademir Vicente Cantarelli,^9^ Alexandre Prehn Zavascki^2,10,11^

^1^ Health Security Partners, Washington, DC, USA

^2^ Infectious Disease Service, Hospital de Clínicas de Porto Alegre, Porto Alegre, RS, Brazil

^3^Occupational Medicine Service, Hospital de Clínicas de Porto Alegre, Porto Alegre, RS, Brazil

^4^Postgraduate Program in Pneumological Sciences, Universidade Federal do Rio Grande do Sul, Porto Alegre, RS, Brazil

^5^Laboratório de Genética e Biologia Molecular, Hospital Moinhos de Vento, RS, Porto Alegre, Brazil

^6^Internal Bed Regulation Committee, Hospital de Clínicas de Porto Alegre, Porto Alegre, RS, Brazil

^7^Unimed Vale dos Sinos, Novo Hamburgo, RS, Brazil

^8^Universidade do Vale do Rio dos Sinos, São Leopoldo, RS, Brazil

^9^Universidade Federal de Ciências da Saúde de Porto Alegre, Porto Alegre, RS, Brazil

^10^Infectious Disease and Infection Control Service, Hospital Moinhos de Vento, RS, Porto Alegre, Brazil

^11^Department of Internal Medicine, Universidade Federal do Rio Grande do Sul, Porto Alegre, RS, Brazil

*Address correspondence to Flávia Roberta Brust: flavia@healthsecuritypartners.org

First Author and Second Author contributed equally to this work. Author order was determined alphabetically.

SUPPLEMENTAL MATERIAL

**Methods used for molecular testing and whole-genome sequencing**

Real-time RT-PCRs were performed by using the commercial kits TaqMan 2019-nCoV Assay Kit v1 (Thermo Fisher Scientific, https://www.thermofisher.com/us), TaqCheck SARS CoV-2 Fast PCR Assay (Thermo Fisher Scientific) or Xpert Xpress SARS-CoV-2 (Cepheid, https://www.cepheid.com/en-US).

The viral genomic library was prepared by using the CleanPlex SARS-CoV-2 FLEX Kit and the SARS-CoV-2 Emerging Variants Panel Add-on v2 (Paragon Genomics, Inc, www.paragongenomics.com) following the manufacturer's instructions. The resulting libraries were sequenced on the Illumina MiSeq platform by using a V2 chemistry (Illumina, https://www.illumina.com).

The FASTq reads were aligned with complete SARS-CoV-2 genomes using CleanPlex SARS hCoV2 pipeline on SOPHiA DDM™ Software or Illumina® DRAGEN COVID Lineage App. SARS-CoV-2 variants were characterized using the NextClade online platform and lineages were identified by Pangolin online platform.

**Supplementary Table**

**Table S1.** Most common SARS-CoV-2 lineages identified in our study and in Brazil, February 2023 to August 2024

**Table S2.** Comparison of the distribution of the 10 most prevalent SARS-CoV-2 lineages between healthcare workers and inpatients

**Table S3.** GISAID accession number of all SARS-CoV-2 genome sequences of healthcare workers and inpatients at Southern Brazil, February 2023 to August 2024

**Supplementary Figure**

**FIG S1.** Flowchart of the study population

**Table S1.** Most frequent SARS-CoV-2 lineages reported in our study and in Brazil, February 2023 to August 2024

| **Month/year** |  | **XBB.1.5** | **FE.1*** | **XBB.2.3** | **XBB.1.5.70.* +GK.1.*** | **JD.1*** | **JN.1.* (%)** | **JN.1.7.*** | **JN.1.16.*** | **KP.2*** | **KP.3*** | **MJ.1*** | **Other** |
| --- | --- | --- | --- | --- | --- | --- | --- | --- | --- | --- | --- | --- | --- |
| **Feb/23** | RS (n=9) | 44.4 |  |  |  |  |  |  |  |  |  |  | 55.6 |
|  | BR | 33.1 |  |  |  |  |  |  |  |  |  |  | 64.2 |
| **Mar/23** | RS (n=53) | 52.8 |  | 26.4 |  |  |  |  |  |  |  |  |  |
|  | BR | 42.8 | 13.4 |  |  |  |  |  |  |  |  |  | 43.8 |
| **Apr/23** | RS (n=63) | 38.1 | 12.7 | 33.3 |  |  |  |  |  |  |  |  |  |
|  | BR | 35.1 | 24.4 |  |  |  |  |  |  |  |  |  | 32.2 |
| **May/23** | RS (n=41) | 36.6 | 26.8 | 17.1 |  |  |  |  |  |  |  |  |  |
|  | BR | 19.9 | 39.8 |  |  |  |  |  |  |  |  |  | 30.8 |
| **Jun/23** | RS (n=8) | 25.0 |  | 25.0 |  |  |  |  |  |  |  |  | 37.5 |
|  | BR |  | 39.3 |  | 20.7 |  |  |  |  |  |  |  | 21.8 |
| **Jul/23** | RS (n=7) | 14.3 | 42.9 | 14.3 | 14.3 |  |  |  |  |  |  |  |  |
|  | BR |  | 31.4 |  | 31.2 |  |  |  |  |  |  |  | 20.0 |
| **Agu/23** | RS (n=12) | 16.7 | 25.0 |  | 33.3 |  |  |  |  |  |  |  |  |
|  | BR |  | 13.1 |  | 43.1 | 16.0 |  |  |  |  |  |  | 20.0 |
| **Sep/23** | RS (n=46) |  |  |  | 71.7 | 13.0 |  |  |  |  |  |  |  |
|  | BR |  |  |  | 46.2 | 22.9 |  |  |  |  |  |  | 20.0 |
| **Oct/23** | RS (n=128) |  |  |  | 55.5 | 22.7 |  |  |  |  |  |  |  |
|  | BR |  |  |  | 44.8 | 41.0 |  |  |  |  |  |  |  |
| **Nov/23** | RS (n=111) |  |  |  | 44.1 | 31.5 |  |  |  |  |  |  |  |
|  | BR |  |  |  | 29.7 | 48.9 | 12.7 |  |  |  |  |  |  |
| **Dec/23** | RS (n=76) |  |  |  | 34.2 | 38.2 | 15.8 |  |  |  |  |  |  |
|  | BR |  |  |  | 16.2 | 31.5 | 46.3 |  |  |  |  |  |  |
| **Jan/24** | RS (n=126) |  |  |  |  | 16.7 | 60.3 |  |  |  |  |  |  |
|  | BR |  |  |  |  | 16.6 | 58.8 |  |  |  |  |  | 16.7 |
| **Feb/24** | RS (n=140) |  |  |  |  |  | 77.1 |  |  |  |  |  |  |
|  | BR |  |  |  |  |  | 71.7 |  |  |  |  |  | 15.8 |
| **Mar/24** | RS (n=126) |  |  |  |  |  | 77.8 |  |  |  |  |  |  |
|  | BR |  |  |  |  |  | 73.9 |  |  |  |  |  | 15.5 |
| **Apr/24** | RS (n=27) |  |  |  |  |  | 55.6 | 22.2 |  |  |  |  |  |
|  | BR |  |  |  |  |  | 66.7 |  |  |  |  |  | 16.2 |
| **May/24** | RS (n=2) |  |  |  |  |  |  | 50.0 |  |  |  |  | 50.0 |
|  | BR |  |  |  |  |  | 66.7 |  |  |  |  |  | 15.7 |
| **Jun/24** | RS (n=2) |  |  |  |  |  | 50.0 |  |  |  |  |  | 50.0 |
|  | BR |  |  |  |  |  | 42.3 |  | 15.1 |  |  |  |  |
| **Jul/24** | RS (n=8) |  |  |  |  |  |  |  |  | 25.0 | 25.0 |  | 50.0 |
|  | BR |  |  |  |  |  | 29.5 |  | 16.3 | 10.2 |  | 15.5 |  |
| **Aug/24** | RS (n=8) |  |  |  |  |  |  |  |  | 12.5 | 12.5 | 12.5 | 62.5 |
|  | BR |  |  |  |  |  | 25.0 |  |  | 19.0 | 16.1 |  |  |

Lineages are represented in percentage; Rio Grande do Sul (RS): most frequent SARS-CoV-2 lineages reported in our study; Brazil (BR): most frequent SARS-CoV-2 lineages reported in Brazil according to the Fiocruz Genomic Surveillance Network (https://www.genomahcov.fiocruz.br/dashboard-en/)

**Table S2.** Comparison of the distribution of the 10 most prevalent SARS-CoV-2 lineages between healthcare workers and inpatients

| **Lineage** | **Healthcare workers** | **Inpatient** | **p_value** | **p_adjusted** |
| --- | --- | --- | --- | --- |
| JN.1 | 133 (17.8%) | 29 (11.9%) | 0.0358 | 0.1791 |
| GK.1.1 | 82 (10.9%) | 33 (13.5%) | 0.2997 | 0.5995 |
| JD.1.1 | 66 (8.8%) | 14 (5.7%) | 0.1376 | 0.4585 |
| XBB.1.5 | 44 (5.9%) | 20 (8.2%) | 0.2290 | 0.5725 |
| JN.1.9 | 48 (6.4%) | 4 (1.6%) | 0.0025* | 0.0250***** |
| XBB.2.3 | 33 (4.4%) | 12 (4.9%) | 0.7247 | 0.9059 |
| JN.1.43.1 | 32 (4.3%) | 9 (3.7%) | 0.8532 | 0.9480 |
| XDR | 25 (3.3%) | 11 (4.5%) | 0.4301 | 0.7168 |
| JD.1.1.1 | 25 (3.3%) | 6 (2.5%) | 0.6717 | 0.9059 |
| GK.1.8 | 20 (2.7%) | 6 (2.5%) | 1.0000 | 1.0000 |

P-values were calculated using Fisher’s exact test and adjusted using the Benjamini-Hochberg method. The overall comparison between groups was statistically significant (p = 0.033). *After multiple testing corrections, only JN.1.9 remained significantly more frequent among healthcare workers than inpatients .
**Table S3.** GISAID accession number of all SARS-CoV-2 genome sequences of healthcare workers and inpatients at Southern Brazil, February 2023 to August 2024

| **Lineage** | **GISAID accession number** |
| --- | --- |
| BA.2.86 | EPI_ISL_19078768 |
| BA.2.86.3 | EPI_ISL_19078715 |
| BN.1.5 | EPI_ISL_17848966 |
| BQ.1+XBB.1 | EPI_ISL_17848810 |
| BQ.1.1 | EPI_ISL_17848812, EPI_ISL_17848813, EPI_ISL_17848817, EPI_ISL_17848809, EPI_ISL_17848848, EPI_ISL_17848893, EPI_ISL_17848889, EPI_ISL_17848913, EPI_ISL_17848897 |
| BQ.1.1+XBB.1.5 | EPI_ISL_17848946 |
| CH.1.1 | EPI_ISL_17848814 |
| EG.1 | EPI_ISL_17848943, EPI_ISL_17848985 |
| EG.5.1 | EPI_ISL_19414737 |
| EG.5.1.1 | EPI_ISL_18829177, EPI_ISL_19078692, EPI_ISL_18829258, EPI_ISL_19078598 |
| EG.5.1.13 | EPI_ISL_18829162 |
| EG.5.1.3 | EPI_ISL_19078709 |
| EG.5.1.6 | EPI_ISL_18828979, EPI_ISL_18830971, EPI_ISL_19078571, EPI_ISL_19078675, EPI_ISL_18829265, EPI_ISL_19078602, EPI_ISL_18950732, EPI_ISL_19078635, EPI_ISL_19078636 |
| EY.1 | EPI_ISL_17848906, EPI_ISL_17848961 |
| FE.1 | EPI_ISL_17848850, EPI_ISL_17848845, EPI_ISL_18829173, EPI_ISL_18829178 |
| FE.1.1 | EPI_ISL_17848894, EPI_ISL_17848909, EPI_ISL_17848944, EPI_ISL_17848900, EPI_ISL_17848920, EPI_ISL_17848928, EPI_ISL_17848924, EPI_ISL_17848927, EPI_ISL_17848970, EPI_ISL_17848978, EPI_ISL_18107193, EPI_ISL_18828991, EPI_ISL_18828980 |
| FE.1.1.4 | EPI_ISL_19078567 |
| FE.1.2 | EPI_ISL_17848892, EPI_ISL_17848983, EPI_ISL_17848940, EPI_ISL_17848933, EPI_ISL_17848965, EPI_ISL_17848971, EPI_ISL_17848972, EPI_ISL_17848974, EPI_ISL_17848975, EPI_ISL_18107191, EPI_ISL_18107194, EPI_ISL_18829267, EPI_ISL_18829277, EPI_ISL_18829282 |
| FH.1 | EPI_ISL_17848883 |
| FK.1.1 | EPI_ISL_17848938 |
| FL.13.5 | EPI_ISL_19078580 |
| FL.4 | EPI_ISL_17848934, EPI_ISL_17848953, EPI_ISL_17848987, EPI_ISL_18107183 |
| GJ.1.2 | EPI_ISL_18828986 |
| GJ.4 | EPI_ISL_19078683 |
| GK.1 | EPI_ISL_18107185, EPI_ISL_18828982, EPI_ISL_18829161, EPI_ISL_18829190, EPI_ISL_19414812, EPI_ISL_19078662, EPI_ISL_19078663, EPI_ISL_19078679, EPI_ISL_19078595, EPI_ISL_18829263, EPI_ISL_18831013, EPI_ISL_18829292, EPI_ISL_18831012, EPI_ISL_18830995, EPI_ISL_19078754, EPI_ISL_19078638, EPI_ISL_19414680 |
| GK.1.1 | EPI_ISL_18828983, EPI_ISL_18828985, EPI_ISL_18828987, EPI_ISL_18828988, EPI_ISL_18951477, EPI_ISL_18829153, EPI_ISL_18829154, EPI_ISL_18828990, EPI_ISL_19414558, EPI_ISL_19414559, EPI_ISL_19158818, EPI_ISL_19158819, EPI_ISL_18829174, EPI_ISL_19158820, EPI_ISL_18829175, EPI_ISL_19158821, EPI_ISL_19158822, EPI_ISL_18829176, EPI_ISL_18829179, EPI_ISL_18829180, EPI_ISL_18829166, EPI_ISL_18829170, EPI_ISL_18829169, EPI_ISL_18829164, EPI_ISL_18829184, EPI_ISL_18829185, EPI_ISL_18829181, EPI_ISL_18829182, EPI_ISL_18829183, EPI_ISL_19433686, EPI_ISL_18829186, EPI_ISL_18829191, EPI_ISL_19158824, EPI_ISL_19158823, EPI_ISL_18829188, EPI_ISL_18829189, EPI_ISL_19078568, EPI_ISL_19078569, EPI_ISL_19414816, EPI_ISL_19158804, EPI_ISL_18830942, EPI_ISL_18830939, EPI_ISL_18830943, EPI_ISL_18830962, EPI_ISL_18830981, EPI_ISL_19414809, EPI_ISL_19158806, EPI_ISL_18830967, EPI_ISL_18830944, EPI_ISL_18830951, EPI_ISL_18830972, EPI_ISL_18830973, EPI_ISL_18830975, EPI_ISL_18830980, EPI_ISL_19414811, EPI_ISL_19414814, EPI_ISL_18950741, EPI_ISL_19078708, EPI_ISL_18950751, EPI_ISL_18950752, EPI_ISL_18950746, EPI_ISL_18950749, EPI_ISL_19078575, EPI_ISL_18830947, EPI_ISL_19078579, EPI_ISL_19078583, EPI_ISL_19078585, EPI_ISL_19078570, EPI_ISL_19078576, EPI_ISL_19414560, EPI_ISL_19078665, EPI_ISL_19078676, EPI_ISL_18950720, EPI_ISL_18950721, EPI_ISL_18950724, EPI_ISL_19158805, EPI_ISL_19078680, EPI_ISL_19078685, EPI_ISL_19078686, EPI_ISL_19078687, EPI_ISL_19078688, EPI_ISL_18829254, EPI_ISL_19078587, EPI_ISL_19078590, EPI_ISL_19078592, EPI_ISL_19078589, EPI_ISL_19078593, EPI_ISL_19078703, EPI_ISL_19078702, EPI_ISL_18950726, EPI_ISL_18829269, EPI_ISL_18829275, EPI_ISL_18829276, EPI_ISL_18829294, EPI_ISL_18829280, EPI_ISL_18829289, EPI_ISL_19414557, EPI_ISL_18829291, EPI_ISL_18829295, EPI_ISL_19078718, EPI_ISL_19078724, EPI_ISL_19078725, EPI_ISL_18830982, EPI_ISL_18830985, EPI_ISL_18830997, EPI_ISL_18830999, EPI_ISL_18831005, EPI_ISL_18831006, EPI_ISL_18831010, EPI_ISL_18831001, EPI_ISL_19078623, EPI_ISL_19078624, EPI_ISL_19078749, EPI_ISL_19078750 |
| GK.1.10 | EPI_ISL_19078674, EPI_ISL_18830959 |
| GK.1.10.1 | EPI_ISL_19078618 |
| GK.1.11.1 | EPI_ISL_19078695, EPI_ISL_19078706, EPI_ISL_19078696, EPI_ISL_19078700, EPI_ISL_19078712, EPI_ISL_19078714 |
| GK.1.2 | EPI_ISL_18830948, EPI_ISL_18830949 |
| GK.1.2.1 | EPI_ISL_19078666, EPI_ISL_18830986, EPI_ISL_18830954 |
| GK.1.4 | EPI_ISL_18829271 |
| GK.1.5 | EPI_ISL_18830940 |
| GK.1.8 | EPI_ISL_18829168, EPI_ISL_19414815, EPI_ISL_18830941, EPI_ISL_18830938, EPI_ISL_18950750, EPI_ISL_19414552, EPI_ISL_19078671, EPI_ISL_19078659, EPI_ISL_19078678, EPI_ISL_19414561, EPI_ISL_19078594, EPI_ISL_18829255, EPI_ISL_19414554, EPI_ISL_19414555, EPI_ISL_19078704, EPI_ISL_19414818, EPI_ISL_18950728, EPI_ISL_19414556, EPI_ISL_19078604, EPI_ISL_18829281, EPI_ISL_18829283, EPI_ISL_19078719, EPI_ISL_18831009, EPI_ISL_19414810, EPI_ISL_19414744, EPI_ISL_18950739 |
| GK.1.8.1 | EPI_ISL_19078610, EPI_ISL_19078612 |
| GK.11 | EPI_ISL_19078668 |
| GK.8 | EPI_ISL_18829165 |
| HK.3.2 | EPI_ISL_18830937, EPI_ISL_19078681, EPI_ISL_19078701, EPI_ISL_19078723, EPI_ISL_18830990, EPI_ISL_18831003, EPI_ISL_19078639, EPI_ISL_19433696 |
| HK.6 | EPI_ISL_18830946, EPI_ISL_19078582, EPI_ISL_19078584, EPI_ISL_19078577, EPI_ISL_18830945 |
| HN.2 | EPI_ISL_18830965 |
| HN.6 | EPI_ISL_18830963, EPI_ISL_19078591, EPI_ISL_18950727 |
| HV.1 | EPI_ISL_18830964, EPI_ISL_18830978, EPI_ISL_18950748, EPI_ISL_19078572, EPI_ISL_18950723, EPI_ISL_19078698, EPI_ISL_19078601, EPI_ISL_18829264, EPI_ISL_18830950, EPI_ISL_18829273, EPI_ISL_18829278, EPI_ISL_18829288, EPI_ISL_19078606, EPI_ISL_18830958, EPI_ISL_19414681 |
| JD.1.1 | EPI_ISL_18829157, EPI_ISL_18829158, EPI_ISL_18829159, EPI_ISL_18829155, EPI_ISL_18829156, EPI_ISL_18830968, EPI_ISL_18830969, EPI_ISL_18830976, EPI_ISL_19414817, EPI_ISL_18830979, EPI_ISL_18950742, EPI_ISL_18950743, EPI_ISL_19078573, EPI_ISL_19078578, EPI_ISL_19078581, EPI_ISL_19078661, EPI_ISL_19078677, EPI_ISL_19078660, EPI_ISL_19078670, EPI_ISL_18950722, EPI_ISL_18950719, EPI_ISL_19414553, EPI_ISL_19078684, EPI_ISL_19414562, EPI_ISL_19078689, EPI_ISL_19078690, EPI_ISL_18830952, EPI_ISL_18829259, EPI_ISL_19158803, EPI_ISL_18950725, EPI_ISL_18829260, EPI_ISL_19414563, EPI_ISL_19078697, EPI_ISL_19078600, EPI_ISL_19078710, EPI_ISL_18829262, EPI_ISL_18829266, EPI_ISL_18829268, EPI_ISL_18829270, EPI_ISL_19078713, EPI_ISL_18829272, EPI_ISL_18829274, EPI_ISL_18829293, EPI_ISL_18830987, EPI_ISL_18829279, EPI_ISL_18829284, EPI_ISL_18829285, EPI_ISL_18829290, EPI_ISL_19078720, EPI_ISL_19078722, EPI_ISL_19078726, EPI_ISL_18830983, EPI_ISL_18830984, EPI_ISL_18830988, EPI_ISL_18830989, EPI_ISL_18830992, EPI_ISL_18830994, EPI_ISL_18831000, EPI_ISL_19078729, EPI_ISL_18831007, EPI_ISL_18831008, EPI_ISL_18831011, EPI_ISL_18831004, EPI_ISL_18830955, EPI_ISL_18830957, EPI_ISL_19078607, EPI_ISL_19078621, EPI_ISL_18831014, EPI_ISL_19078625, EPI_ISL_19078731, EPI_ISL_19078616, EPI_ISL_19078743, EPI_ISL_19414686, EPI_ISL_19078634, EPI_ISL_19414700, EPI_ISL_18950758, EPI_ISL_19414738, EPI_ISL_19414587, EPI_ISL_19433687, EPI_ISL_19414644 |
| JD.1.1.1 | EPI_ISL_18829171, EPI_ISL_18829187, EPI_ISL_18830960, EPI_ISL_18830974, EPI_ISL_19078586, EPI_ISL_19078673, EPI_ISL_18829257, EPI_ISL_19078599, EPI_ISL_18829256, EPI_ISL_19078588, EPI_ISL_19078603, EPI_ISL_18830996, EPI_ISL_19078728, EPI_ISL_19078605, EPI_ISL_19078609, EPI_ISL_19078620, EPI_ISL_19078744, EPI_ISL_19078745, EPI_ISL_19078746, EPI_ISL_19414674, EPI_ISL_19078747, EPI_ISL_19414677, EPI_ISL_19078748, EPI_ISL_18950756, EPI_ISL_19414729, EPI_ISL_19414732, EPI_ISL_19447424, EPI_ISL_19078776, EPI_ISL_18950740, EPI_ISL_19414589, EPI_ISL_19078785 |
| JD.1.1.6 | EPI_ISL_19078705, EPI_ISL_19078711 |
| JD.1.1.7 | EPI_ISL_19078682 |
| JD.1.1.8 | EPI_ISL_18950744, EPI_ISL_18950747, EPI_ISL_19078664, EPI_ISL_19078667, EPI_ISL_19078669, EPI_ISL_18950718, EPI_ISL_19078693, EPI_ISL_18950729, EPI_ISL_19078716, EPI_ISL_19078721, EPI_ISL_19078619, EPI_ISL_18950733, EPI_ISL_19078613, EPI_ISL_19414573, EPI_ISL_18950738, EPI_ISL_18950755 |
| JD.1.2 | EPI_ISL_19078657 |
| JN.1 | EPI_ISL_18830961, EPI_ISL_18829261, EPI_ISL_18829251, EPI_ISL_18829252, EPI_ISL_18829253, EPI_ISL_18829249, EPI_ISL_18829250, EPI_ISL_19078694, EPI_ISL_19078699, EPI_ISL_18829286, EPI_ISL_18829287, EPI_ISL_19078727, EPI_ISL_18830991, EPI_ISL_18830993, EPI_ISL_18830998, EPI_ISL_18831002, EPI_ISL_18830953, EPI_ISL_18830956, EPI_ISL_19078622, EPI_ISL_19078611, EPI_ISL_18831015, EPI_ISL_19078734, EPI_ISL_19078735, EPI_ISL_19078737, EPI_ISL_19078738, EPI_ISL_19078617, EPI_ISL_19078626, EPI_ISL_19078628, EPI_ISL_19078741, EPI_ISL_19078742, EPI_ISL_19078629, EPI_ISL_19078630, EPI_ISL_19078656, EPI_ISL_19078640, EPI_ISL_19414679, EPI_ISL_19078631, EPI_ISL_19078637, EPI_ISL_19078633, EPI_ISL_19414693, EPI_ISL_19414694, EPI_ISL_19414701, EPI_ISL_18950754, EPI_ISL_19078644, EPI_ISL_19078643, EPI_ISL_19414581, EPI_ISL_18950734, EPI_ISL_19414715, EPI_ISL_19414722, EPI_ISL_19078645, EPI_ISL_19078650, EPI_ISL_19078655, EPI_ISL_19078651, EPI_ISL_19078652, EPI_ISL_18950735, EPI_ISL_19414592, EPI_ISL_19078772, EPI_ISL_18950759, EPI_ISL_19078773, EPI_ISL_19414741, EPI_ISL_19414580, EPI_ISL_19414579, EPI_ISL_19078777, EPI_ISL_19078756, EPI_ISL_19078755, EPI_ISL_19078758, EPI_ISL_19078760, EPI_ISL_18950761, EPI_ISL_19078761, EPI_ISL_19078762, EPI_ISL_19078764, EPI_ISL_18950763, EPI_ISL_19078778, EPI_ISL_19078779, EPI_ISL_19078765, EPI_ISL_19078767, EPI_ISL_19414596, EPI_ISL_19078770, EPI_ISL_19414600, EPI_ISL_19414591, EPI_ISL_19078780, EPI_ISL_19078781, EPI_ISL_19078782, EPI_ISL_19416594, EPI_ISL_19414620, EPI_ISL_19433688, EPI_ISL_19078783, EPI_ISL_19078784, EPI_ISL_19416595, EPI_ISL_19414766, EPI_ISL_19414599, EPI_ISL_19078788, EPI_ISL_19078789, EPI_ISL_19433689, EPI_ISL_19078790, EPI_ISL_19433693, EPI_ISL_19433694, EPI_ISL_19433695, EPI_ISL_19433697, EPI_ISL_19433698, EPI_ISL_19433699, EPI_ISL_19414767, EPI_ISL_19414768, EPI_ISL_19414769, EPI_ISL_19414597, EPI_ISL_19414594, EPI_ISL_19433702, EPI_ISL_19433704, EPI_ISL_19433705, EPI_ISL_19433706, EPI_ISL_19433707, EPI_ISL_19433708, EPI_ISL_19433709, EPI_ISL_19433711, EPI_ISL_19433712, EPI_ISL_19414601, EPI_ISL_19414602, EPI_ISL_19414603, EPI_ISL_19414622, EPI_ISL_19414613, EPI_ISL_19433713, EPI_ISL_19414748, EPI_ISL_19414751, EPI_ISL_19414754, EPI_ISL_19414755, EPI_ISL_19414758, EPI_ISL_19414760, EPI_ISL_19414615, EPI_ISL_19414629, EPI_ISL_19414638, EPI_ISL_19433716, EPI_ISL_19433718, EPI_ISL_19414763, EPI_ISL_19414764, EPI_ISL_19414634, EPI_ISL_19414635, EPI_ISL_19414643, EPI_ISL_19414775, EPI_ISL_19414778, EPI_ISL_19414780, EPI_ISL_19414781, EPI_ISL_19414641, EPI_ISL_19414784, EPI_ISL_19414785, EPI_ISL_19447431, EPI_ISL_19414797, EPI_ISL_19447426, EPI_ISL_19414791, EPI_ISL_19414793, EPI_ISL_19414799, EPI_ISL_19447406, EPI_ISL_19414651, EPI_ISL_19414650, EPI_ISL_19414668, EPI_ISL_19414653, EPI_ISL_19414660, EPI_ISL_19414659, EPI_ISL_19414658, EPI_ISL_19414666, EPI_ISL_19447407, EPI_ISL_19447429, EPI_ISL_19414671, EPI_ISL_19447416 |
| JN.1.1 | EPI_ISL_18831016, EPI_ISL_19078615, EPI_ISL_19078642, EPI_ISL_19078654, EPI_ISL_19078649, EPI_ISL_19078653, EPI_ISL_18950736, EPI_ISL_19078774, EPI_ISL_19414571, EPI_ISL_19078786, EPI_ISL_19414618 |
| JN.1.13 | EPI_ISL_19433710, EPI_ISL_19414752 |
| JN.1.16 | EPI_ISL_19414595, EPI_ISL_19433701, EPI_ISL_19414657, EPI_ISL_19447408, |
| JN.1.18 | EPI_ISL_19078769 |
| JN.1.2 | EPI_ISL_19414719 |
| JN.1.21 | EPI_ISL_19078763 |
| JN.1.22 | EPI_ISL_19078608, EPI_ISL_19078641, EPI_ISL_19414712, EPI_ISL_19414743, EPI_ISL_19414574, EPI_ISL_19078759, EPI_ISL_18950762, EPI_ISL_19078787, EPI_ISL_19414770, EPI_ISL_19414608, EPI_ISL_19414773, EPI_ISL_18829165, EPI_ISL_19433715 |
| JN.1.23 | EPI_ISL_19433691, EPI_ISL_19414633 |
| JN.1.29 | EPI_ISL_19414678, EPI_ISL_19414569, EPI_ISL_19414772, EPI_ISL_19414759 |
| JN.1.32 | EPI_ISL_19414654 |
| JN.1.39 | EPI_ISL_19414665 |
| JN.1.4 | EPI_ISL_19078732, EPI_ISL_19078751, EPI_ISL_19078739, EPI_ISL_19414695, EPI_ISL_19078775, EPI_ISL_19433692, EPI_ISL_19414748, EPI_ISL_19414626, EPI_ISL_19447404, EPI_ISL_19414788 |
| JN.1.4.2 | EPI_ISL_19078614, EPI_ISL_18950753, EPI_ISL_19078771, EPI_ISL_19414765, EPI_ISL_19078791, EPI_ISL_19414614, EPI_ISL_19414790 |
| JN.1.4.5 | EPI_ISL_19414684 |
| JN.1.43.1 | EPI_ISL_19414673, EPI_ISL_19414687, EPI_ISL_19414698, EPI_ISL_19414707, EPI_ISL_19414708, EPI_ISL_19414567, EPI_ISL_19414583, EPI_ISL_19414584, EPI_ISL_19414572, EPI_ISL_19414745, EPI_ISL_19414747, EPI_ISL_19447403, EPI_ISL_19447425, EPI_ISL_19433700, EPI_ISL_19414598, EPI_ISL_19414607, EPI_ISL_19414605, EPI_ISL_19414662, EPI_ISL_19414748, EPI_ISL_19414750, EPI_ISL_19414761, EPI_ISL_19414617, EPI_ISL_19414616, EPI_ISL_19414649, EPI_ISL_19414627, EPI_ISL_19414628, EPI_ISL_19414631, EPI_ISL_19414774, EPI_ISL_19414625, EPI_ISL_19414777, EPI_ISL_19414779, EPI_ISL_19414794, EPI_ISL_19414800, EPI_ISL_19414787, EPI_ISL_19414795, EPI_ISL_19414796, EPI_ISL_19416597, EPI_ISL_19447409, EPI_ISL_19414663, EPI_ISL_19414655, EPI_ISL_19414669 |
| JN.1.5 | EPI_ISL_19078648 |
| JN.1.55 | EPI_ISL_19414792 |
| JN.1.58 | EPI_ISL_19414609, EPI_ISL_19414623, EPI_ISL_19414630, EPI_ISL_19414624, EPI_ISL_19414639, EPI_ISL_19414640, EPI_ISL_19414636 |
| JN.1.61 | EPI_ISL_19414789, EPI_ISL_19414667 |
| JN.1.7 | EPI_ISL_19414735, EPI_ISL_19414819, EPI_ISL_19078766, EPI_ISL_19433690, EPI_ISL_19414762, EPI_ISL_19466060, EPI_ISL_19414783, EPI_ISL_19414798, EPI_ISL_19414656, EPI_ISL_19447412, EPI_ISL_19447415, EPI_ISL_19447411, EPI_ISL_19447433, EPI_ISL_19447434, EPI_ISL_19447437 |
| JN.1.7.2 | EPI_ISL_19414566, EPI_ISL_19414740, EPI_ISL_19459944, EPI_ISL_19459945, EPI_ISL_19459946, EPI_ISL_19416596, EPI_ISL_19414786, EPI_ISL_19414645, EPI_ISL_19414646, EPI_ISL_19414647, EPI_ISL_19447405, EPI_ISL_19447414 |
| JN.1.8.1 | EPI_ISL_19414652, EPI_ISL_19447413 |
| JN.1.9 | EPI_ISL_19414682, EPI_ISL_19414685, EPI_ISL_19414688, EPI_ISL_19414689, EPI_ISL_19414690, EPI_ISL_19414691, EPI_ISL_19414692, EPI_ISL_19414696, EPI_ISL_19414697, EPI_ISL_19414702, EPI_ISL_19414703, EPI_ISL_19414705, EPI_ISL_19414706, EPI_ISL_19414709, EPI_ISL_19414710, EPI_ISL_19414713, EPI_ISL_19414714, EPI_ISL_19414716, EPI_ISL_19414717, EPI_ISL_19414718, EPI_ISL_19414720, EPI_ISL_19414721, EPI_ISL_19414723, EPI_ISL_19414724, EPI_ISL_19414725, EPI_ISL_19414726, EPI_ISL_19414728, EPI_ISL_19414730, EPI_ISL_19414731, EPI_ISL_19414733, EPI_ISL_19414734, EPI_ISL_19414568, EPI_ISL_19414736, EPI_ISL_19414739, EPI_ISL_19414742, EPI_ISL_19414570, EPI_ISL_19414746, EPI_ISL_19414578, EPI_ISL_19414575, EPI_ISL_19414577, EPI_ISL_19414576, EPI_ISL_19414586, EPI_ISL_19414585, EPI_ISL_19414588, EPI_ISL_19414593, EPI_ISL_19414590, EPI_ISL_19414610, EPI_ISL_19414611, EPI_ISL_19414753, EPI_ISL_19414672, EPI_ISL_19447410, EPI_ISL_19414670 |
| JN.2.5 | EPI_ISL_19078740, EPI_ISL_19447435 |
| KA.1 | EPI_ISL_19078717 |
| KK.1 | EPI_ISL_19078597 |
| KP.2.3 | EPI_ISL_19447417, EPI_ISL_19447423, EPI_ISL_19447490 |
| KP.3.2.4 | EPI_ISL_19447421 |
| KQ.1 | EPI_ISL_19414612 |
| KR.1 | EPI_ISL_19414756, EPI_ISL_19433717 |
| LE.1 | EPI_ISL_19414664, EPI_ISL_19447427, EPI_ISL_19447428, EPI_ISL_19447430, EPI_ISL_19447436 |
| LL.1 | EPI_ISL_19414776 |
| LQ.1.1 | EPI_ISL_19447420, EPI_ISL_19447418, EPI_ISL_19447419, EPI_ISL_19447422 |
| MC.13 | EPI_ISL_19447492 |
| XBB.1.15 | EPI_ISL_17848895 |
| XBB.1.16 | EPI_ISL_18830977 |
| XBB.1.16.15 | EPI_ISL_18951476 |
| XBB.1.16.6 | EPI_ISL_18950745 |
| XBB.1.16.9 | EPI_ISL_18829160 |
| XBB.1.18 | EPI_ISL_17848896, EPI_ISL_17848968, EPI_ISL_18107187 |
| XBB.1.18.1 | EPI_ISL_17848829, EPI_ISL_17848837, EPI_ISL_17848838, EPI_ISL_17848904 |
| XBB.1.4.2 | EPI_ISL_18107188, EPI_ISL_18107190 |
| XBB.1.5 | EPI_ISL_17848811, EPI_ISL_17848815, EPI_ISL_17848816, EPI_ISL_17848819, EPI_ISL_17848818, EPI_ISL_17848830, EPI_ISL_17848832, EPI_ISL_17848834, EPI_ISL_17848836, EPI_ISL_17848856, EPI_ISL_17848839, EPI_ISL_17848852, EPI_ISL_17848853, EPI_ISL_17848841, EPI_ISL_17848914, EPI_ISL_17848846, EPI_ISL_17848867, EPI_ISL_17848857, EPI_ISL_17848860, EPI_ISL_17848957, EPI_ISL_17848861, EPI_ISL_17848862, EPI_ISL_17848863, EPI_ISL_17848865, EPI_ISL_18103846, EPI_ISL_17848866, EPI_ISL_17848902, EPI_ISL_17848881, EPI_ISL_17848907, EPI_ISL_17848905, EPI_ISL_17848882, EPI_ISL_18107178, EPI_ISL_17848899, EPI_ISL_17848901, EPI_ISL_17848963, EPI_ISL_17848912, EPI_ISL_17848988, EPI_ISL_17848960, EPI_ISL_17848917, EPI_ISL_17848918, EPI_ISL_17848935, EPI_ISL_17848916, EPI_ISL_17848948, EPI_ISL_17848942, EPI_ISL_17848949, EPI_ISL_17848929, EPI_ISL_17848921, EPI_ISL_17848922, EPI_ISL_17848932, EPI_ISL_17848931, EPI_ISL_17848951, EPI_ISL_17848967, EPI_ISL_17848952, EPI_ISL_17848925, EPI_ISL_17848976, EPI_ISL_17848980, EPI_ISL_17848981, EPI_ISL_18107192, EPI_ISL_18828978, EPI_ISL_18828989, EPI_ISL_19078691, EPI_ISL_19078707, EPI_ISL_18950731, EPI_ISL_19078646 |
| XBB.1.5.1 | EPI_ISL_18103848 |
| XBB.1.5.13 | EPI_ISL_17848923 |
| XBB.1.5.14 | EPI_ISL_17848973 |
| XBB.1.5.15 | EPI_ISL_18103847, EPI_ISL_17848939, EPI_ISL_17848984 |
| XBB.1.5.18.1 | EPI_ISL_17848910, EPI_ISL_17848936, EPI_ISL_17848937 |
| XBB.1.5.19 | EPI_ISL_17848864, EPI_ISL_17848873, EPI_ISL_18107179 |
| XBB.1.5.28 | EPI_ISL_17848859 |
| XBB.1.5.40 | EPI_ISL_17848964 |
| XBB.1.5.59 | EPI_ISL_17848977. EPI_ISL_17848982, EPI_ISL_18828984 |
| XBB.1.5.70 | EPI_ISL_18107182, EPI_ISL_18107184, EPI_ISL_18828981, EPI_ISL_18829167, EPI_ISL_18829172, EPI_ISL_18830966, EPI_ISL_18830970, EPI_ISL_19158807, EPI_ISL_19158808, EPI_ISL_19078574, EPI_ISL_19078672, EPI_ISL_18950730, EPI_ISL_19078596 |
| XBB.1.9.1 | EPI_ISL_17848835, EPI_ISL_17848849 |
| XBB.2.3 | EPI_ISL_17848820, EPI_ISL_17848831, EPI_ISL_17848833, EPI_ISL_17848851, EPI_ISL_17848840, EPI_ISL_17848842, EPI_ISL_17848843, EPI_ISL_17848854, EPI_ISL_17848844, EPI_ISL_17848858, EPI_ISL_17848956, EPI_ISL_17848891, EPI_ISL_17848855, EPI_ISL_17848903, EPI_ISL_17848955, EPI_ISL_17848954, EPI_ISL_17848890, EPI_ISL_17848884, EPI_ISL_17848885, EPI_ISL_17848886, EPI_ISL_17848887, EPI_ISL_17848908, EPI_ISL_17848898, EPI_ISL_17848911, EPI_ISL_18107181, EPI_ISL_17848888, EPI_ISL_17848962, EPI_ISL_18107180 EPI_ISL_17848945, EPI_ISL_17848958, EPI_ISL_17848959, EPI_ISL_17848926, EPI_ISL_17848941, EPI_ISL_17848950, EPI_ISL_17848919, EPI_ISL_17848930, EPI_ISL_17848915, EPI_ISL_17848969, EPI_ISL_17848947, EPI_ISL_17848986, EPI_ISL_17848989, EPI_ISL_17848979, EPI_ISL_18107186, EPI_ISL_18103849, EPI_ISL_18107189 |
| XBB.2.4 | EPI_ISL_19078658 |
| XCH.1 | EPI_ISL_18829163 |
| XDP | EPI_ISL_19414582 |
| XDR | EPI_ISL_19078730, EPI_ISL_18950737, EPI_ISL_19078733, EPI_ISL_19078736, EPI_ISL_19078752, EPI_ISL_19078627, EPI_ISL_18950760, EPI_ISL_19414675, EPI_ISL_19414676, EPI_ISL_19414683, EPI_ISL_19078753, EPI_ISL_19078632, EPI_ISL_19414699, EPI_ISL_19414704, EPI_ISL_19414711, EPI_ISL_19414727, EPI_ISL_18950757, EPI_ISL_19416398, EPI_ISL_19078756, EPI_ISL_19078647, EPI_ISL_19078757, EPI_ISL_19414621, EPI_ISL_19433703, EPI_ISL_19414606, EPI_ISL_19414604, EPI_ISL_19414619, EPI_ISL_19414749, EPI_ISL_19414757, EPI_ISL_19414632, EPI_ISL_19414782, EPI_ISL_19414642, EPI_ISL_19414637, EPI_ISL_19447432, EPI_ISL_19414801, EPI_ISL_19414648, EPI_ISL_19414659 |

**FIG S1.** Flowchart of the study population
